# The risk of hospitalized infection following initiation of biologic agents versus methotrexate in the treatment of juvenile idiopathic arthritis

**DOI:** 10.1186/s13075-016-1109-8

**Published:** 2016-09-22

**Authors:** Timothy Beukelman, Fenglong Xie, John W. Baddley, Lang Chen, Melissa L. Mannion, Kenneth G. Saag, Jie Zhang, Jeffrey R. Curtis

**Affiliations:** The University of Alabama at Birmingham, 1720 2nd Ave S, Birmingham, AL 35233 USA

## Abstract

**Background:**

In the present study, we compared the incidence of hospitalized infection among children with juvenile idiopathic arthritis (JIA) following initiation of treatment with biologic agents versus methotrexate (MTX).

**Methods:**

We used national Medicaid claims data from 2000 through 2010 to create cohorts of children with JIA who were new users of tumor necrosis factor inhibitors (TNFi), anakinra, and MTX (without concurrent biologic agent use) as defined by a 6-month baseline period of nonuse. Because most anakinra users have systemic juvenile idiopathic arthritis (SJIA), we used claims to identify MTX users who likely had SJIA. Among TNFi users, concurrent MTX use was a time-varying covariate. The study outcome was a primary hospital discharge diagnosis of infection. We calculated adjusted hazard ratios (aHRs) to compare infection rates between biologic agents and MTX.

**Results:**

We identified 3075 new MTX users (160 with SJIA), 2713 new TNFi users, and 247 new anakinra users. There was no increased risk of infection associated with TNFi monotherapy versus MTX (aHR 1.19, 95 % CI 0.72–1.94) or with TNFi + MTX combination therapy versus MTX (aHR 1.23, 95 % CI 0.69–2.17). Baseline high-dose oral glucocorticoid use (≥10 mg/day of prednisone) was associated with infection (aHR 2.03 [95 % CI 1.21–3.39] versus no oral glucocorticoid). Anakinra was associated with infection versus MTX (aHR 3.53 95 % CI 1.83–6.82), but less so compared with MTX users with SJIA (aHR 2.69, 95 % CI 0.82–8.82).

**Conclusions:**

Neither TNFi monotherapy nor TNFi + MTX combination therapy was significantly associated with hospitalized infection compared with MTX. Anakinra was significantly associated with infection, but there was likely residual confounding by disease phenotype.

## Background

Biologic agents, especially tumor necrosis factor inhibitors (TNFi), are widely used in the treatment of juvenile idiopathic arthritis (JIA), and the frequency of their use continues to increase [1–3]. All therapeutic agents are associated with risks, and serious infections are the most commonly occurring serious adverse events in JIA that are possibly caused by biologic agents. Assessing and contextualizing the risk of infection due to use of biologic agents is complicated by the facts that the disease process of JIA itself likely increases the rate of infection [4] and that active JIA must be treated with other systemic immunosuppression, if not with biologic agents, to prevent permanent disability [1]. Thus, there is a clear need for comparative studies of the relative safety of biologic agents in JIA. Despite the frequent use of biologic agents and the need for comparative studies, only a few such studies have been published to date [5, 6].

Among published comparative studies, some do not suggest a significant difference between infection rates associated with TNFi versus methotrexate (MTX) [4, 7], while others do suggest an increased rate associated with TNFi [8, 9]. Similar to the situation with studies of adults with rheumatoid arthritis in which investigators have reported discrepant results, these differences are likely attributable to variations in study populations and study designs [10]. For example, in our previously published study in which we reported no increased risk of infection with TNFi versus MTX, we used a prevalent-user design rather than a methodologically superior new-user design that was not feasible, owing to limited available data at the time [4, 11].

Current JIA treatment recommendations call for the addition of TNFi to MTX (rather than TNFi monotherapy) owing to the demonstrated increased effectiveness of this approach [1]. Nevertheless, many children with JIA are treated with TNFi monotherapy [2]. Importantly, the relative safety of combination therapy versus monotherapy is unclear. The rate of infection associated with TNFi + MTX combination therapy was not increased versus TNFi monotherapy in two published observational studies conducted outside the United States [8, 12], but this issue has not been fully assessed in other studies.

There are several non-TNFi biologic agents currently used for the treatment of JIA, including abatacept, anakinra, canakinumab, and tocilizumab [1, 13]. The relative risk of infection with these biologic agents in JIA is not known [5, 6]. The interleukin (IL)-1 inhibitors anakinra and canakinumab are currently used almost exclusively to treat systemic juvenile idiopathic arthritis (SJIA) [1, 3, 13, 14]. Limited reports of infections associated with anakinra suggest a possible increased risk of infection, but there are no published comparative studies [5]. Importantly, SJIA has a pathogenesis and treatment approach different from those for the other categories of JIA, including the more frequent use of systemic glucocorticoids (GCs) at higher doses [13, 15, 16]. Very little is known about the risks of infection associated with SJIA and its treatment in clinical practice.

In an attempt to address these knowledge gaps, we used national U.S. Medicaid administrative claims data to compare rates of hospitalized infection among children with JIA who were newly starting biologic agents versus those newly starting MTX without concurrent biologic agent use.

## Methods

### Data source

We obtained local institutional review board approval. We conducted this study using Medicaid Analytic eXtract (MAX) files from all 50 U.S. states and the District of Columbia from 2000 through 2010, inclusive. These were the most recent data available to us at the time of the study. MAX files contain medical and pharmacy administrative claims records for low-income children enrolled in Medicaid (U.S. government medical assistance). We have previously published studies of JIA using this data source [4, 17].

### Study cohorts

Using pharmacy and infusion claims, we identified patients who were new users of MTX or biologic agents, including TNFi (etanercept, adalimumab, infliximab, golimumab, and certolizumab), abatacept, anakinra, canakinumab, and tocilizumab. New use was defined by a 6-month baseline clean period of nonuse during which the patient had full medical and pharmacy benefits. Additionally, new users of any TNFi had no use of any TNFi or any other biologic agent during the baseline period (i.e., those switching biologic agents were excluded). Only the first observed use of each medication was considered for new use. Individual patients could have more than one episode of new use during the study period (e.g., MTX and then TNFi). New users who had at least one physician diagnosis code consistent with JIA (International Classification of Diseases, Ninth Revision [ICD-9], codes 714, 696.0, 720) before age 16 years and prior to the new-use prescription fill date (index date) were included. We excluded patients who had any of the following at any time prior to the index date: (1) a physician diagnosis code for JIA when less than 6 months of age; (2) any physician diagnosis code or hospital discharge diagnosis code for inflammatory bowel disease (ICD-9 codes 555, 556); (3) any physician diagnosis code or hospital discharge diagnosis code for malignancy, organ transplant, or HIV infection; and (4) two or more physician diagnosis codes or hospital discharge diagnosis codes for other rheumatologic diseases (systemic lupus erythematosus and other diffuse connective tissue diseases, vasculitis, sarcoidosis) that were at least 7 days but more than 183 days apart. New MTX users were excluded if they used biologic agents at any time prior to the index date.

In order to better evaluate relative infection rates associated with the biologic agents used to treat SJIA, we attempted to identify patients within the MTX new-user cohort who were very likely to have SJIA. However, within ICD-9 there is no specific, reliable physician diagnosis code for SJIA. Therefore, we considered new users of MTX who met any of the following at any time in the data to have SJIA: (1) any physician diagnosis code or hospital discharge diagnosis code for macrophage activation syndrome (ICD-9 code 288.4); (2) any receipt of anakinra, canakinumab, or rilonacept following receipt of MTX; (3) any receipt of cyclosporine in the absence of any physician diagnosis code for uveitis; and (4) any receipt of thalidomide or lenalidomide. Among children with JIA, the development of macrophage activation syndrome [18] and use of the selected medications mentioned above [1, 13] occur nearly exclusively in children with SJIA.

### Medication exposures

The baseline mean daily GC dose was determined by summing the total cumulative oral GC dose dispensed during the 6-month baseline period in milligrams of prednisone equivalents and dividing by 183 days. Baseline mean daily GC dose was categorized as none, low dose (<10 mg of prednisone equivalents per day), and high dose (≥10 mg of prednisone equivalents per day). Among new TNFi users, concurrent MTX use was determined on a time-varying basis during study follow-up. The resultant medication exposure categories were TNFi monotherapy and TNFi + MTX combination therapy, and patients changed exposure categories during follow-up on the basis of their treatment received. Owing to the small number of observed outcomes, we did not adjust or restrict for concurrent MTX use among new anakinra users. The concurrent use of cyclosporine was determined on a time-varying basis during follow-up.

### Study follow-up

Follow-up began on the index date. Follow-up was censored at the first date that any of the following occurred: (1) prescription for corresponding medication cohort (MTX or specific biologic agent) not filled within 90 days of the days supplied by the previous filled prescription or typical infusion interval (e.g., 56 days for infliximab), (2) meeting any of the study cohort exclusion criteria, (3) experiencing the primary study outcome of hospitalized infection, or (4) end of the study period. Patients in the new MTX user cohort were immediately censored if they received a biologic agent.

### Covariates

Sex, race, and age at index date were determined. The presence of physician diagnosis codes or hospital discharge diagnosis codes for the comorbid conditions of psoriasis, diabetes mellitus, and asthma during the 6-month baseline period was assessed. The occurrence of infection during the 6-month baseline period was assessed and assigned to one of three hierarchical levels: none, nonprimary hospital discharge diagnosis or outpatient physician diagnosis code, primary hospital discharge diagnosis (i.e., a nonprimary discharge or outpatient diagnosis was not considered in the presence of a primary hospital discharge diagnosis). Baseline GC use and time-varying concurrent cyclosporine use during follow-up were modeled as covariates.

### Study outcome

The study outcome was a primary hospital discharge diagnosis of infection. The outcome included ICD-9 codes reflecting infections by all organisms (e.g., bacteria, viruses, fungi). As part of sensitivity analyses, we included any hospital discharge diagnosis of infection (i.e., primary and nonprimary hospital discharge diagnoses). The list of infection ICD-9 codes was adapted from previously validated lists [19–21].

### Analyses

We determined the baseline characteristics of the patients in each new medication use cohort. We did not perform statistical tests of the differences between the cohorts, because individual patients could contribute to multiple cohorts and the MTX with SJIA cohort was a subset of the MTX cohort.

We determined crude infection rates for each new medication user cohort if there were at least ten observed infection outcomes. We considered all TNFi inhibitors together as a group compared with MTX in further analyses.

We used Cox proportional hazards regression models to compare the incidence of infections between new biologic agent users and new MTX users. For new users of TNFi, we compared TNFi monotherapy and TNFi + MTX combination therapy versus MTX within the same model. For the new users of anakinra, we separately compared ANA versus MTX and ANA versus MTX with SJIA. We calculated adjusted hazard ratios (aHRs) by using multivariable models to adjust for covariates that were potential confounders of the association between the medication exposures and the outcome. Because individual patients could contribute to more than one new-user cohort, a sandwich variance estimator was applied to account for additional correlations in the data [22]. To evaluate the possibility of statistical interaction between oral GC and biologic agent use and the association with hospitalized infection, we evaluated separate multivariable hazard models for children with and without use of oral GCs during the 6-month baseline period.

We analyzed infection rates among individual TNFi with etanercept as the referent when there were at least ten infection outcomes present. We compared patients’ characteristics among the individual TNFi using chi-square tests. In the multivariable Cox regression models for the comparison of individual TNFi, we additionally adjusted for the number of prior biologic agents used (more than 6 months prior to the index date), and we ignored the concurrent use of MTX.

To evaluate the robustness of our primary results, we performed the following sensitivity analyses: expanded the outcome definition to include hospital discharge diagnosis for infection in any position (i.e., included nonprimary discharge diagnoses), decreased the infection risk window following the days supplied by the last prescription from 90 days to 30 days, decreased the time period used to calculate the baseline mean daily oral GC dose from 183 days to 60 days, and censored all follow-up at 6 months following the index date.

## Results

The patients’ characteristics at the time of newly starting MTX, TNFi, or anakinra are shown in Table 1. There was an insufficient number of infection outcomes to report on new users of abatacept, canakinumab, or tocilizumab. The characteristics of new MTX users with SJIA are also shown. Patients in the MTX with SJIA subcohort met our definition because of any use of cyclosporine without uveitis (55 %), future use of IL-1 inhibitor (51 %), diagnosis of macrophage activation syndrome (10 %), or any use of thalidomide or lenalidomide (8 %).

**Table 1:** Characteristics of patients at the time of medication initiation

	MTX	All TNFi	Anakinra	MTX with SJIA
Number of patients	3075	2713	247	160
Mean age, years (SD)	9.0 (4.7)	10.9 (4.7)	9.4 (4.8)	7.4 (4.6)
Median age (IQR)	9.0 (5.0–13.0)	11.0 (7.0–15.0)	9.0 (6.0–13.0)	6.5 (3.0–11.0)
Female sex, %	66.0	68.3	64.4	56.9
Asthma, %	8.2	6.1	6.5	7.5
Diabetes mellitus, %	1.1	1.2	1.6	0.6
Psoriasis, %	2.7	3.9	0	8.8
Baseline oral GC dose, %				
None	67.1	57.5	22.3	40.0
Low (<10 mg/day)	24.1	29.7	33.6	26.3
High (≥10 mg/day)	8.8	12.9	44.1	33.8
MTX use on index date, %	N/A	47.8	37.3	N/A
Cyclosporine use on index date, %	0.4	1.0	3.6	6.9
Baseline infection, %				
None	47.7	55.8	45.3	35.0
Nonprimary hospitalized or outpatient	49.6	42.1	47.4	54.4
Primary hospitalized	2.7	2.1	7.3	10.6

*Abbreviations: MTX* Methotrexate, *TNFi* Tumor necrosis factor inhibitor, *SJIA* Systemic juvenile idiopathic arthritis, *GC* glucocorticoid, *mg* Milligrams of prednisone equivalent, *N/A* Not applicable

There were clear differences between the cohorts. Specifically, the TNFi users were older, and the anakinra users had higher baseline GC use and more frequent primary hospitalized infections during the baseline period. The MTX users with SJIA were more similar to the anakinra new users with respect to GC use and baseline infections than were all MTX users.

Table 2 shows the crude infection rates for each of the medication cohorts. The crude infection rates for all MTX users, TNFi monotherapy, and TNFi + MTX combination therapy were similar, and ranged from 1.46 to 1.74 infections per 100 person-years. The infection rate for anakinra users was markedly higher at 8.41 infections per 100 person-years. The infection rate for MTX with SJIA was higher than for all MTX users at 2.64 per 100 person-years.

**Table 2:** Crude infection rates for each medication initiation cohort

Medication exposure	Person-years of follow-up	Number of infections	Infection rate per 100 person-years (95 % CI)
MTX	2668.5	39	1.46 (1.07–2.00)
TNFi monotherapy	2144.0	33	1.54 (1.09–2.17)
TNFi + MTX combination	1094.5	19	1.74 (1.11–2.72)
Anakinra with or without MTX	225.9	19	8.41 (5.36–13.2)
MTX with SJIA	113.7	3	2.64 (0.85–8.18)
Etanercept	2594.0	37	1.43 (1.03–1.97)
Adalimumab	413.2	12	2.90 (1.65–5.11)
Infliximab	227.1	3	1.32 (0.43–4.10)

*MTX* Methotrexate, *TNFi* Tumor necrosis factor inhibitor, *SJIA* Systemic juvenile idiopathic arthritis

The most commonly observed types of hospitalized infection overall were pneumonia (19 %), bladder and kidney (13 %), and cellulitis and abscess (10 %). The frequency distribution of the types of infection was not significantly different among new users of MTX, TNFi, and anakinra (data not shown). We observed two hospitalized infections that could be considered opportunistic infections: one case of herpes zoster in a patient receiving TNFi and one case of coccidioidal meningitis in a patient receiving MTX (without prior observed TNFi use).

Table 3 shows the relative hazards of infection in the analysis of TNFi versus MTX. There was not a significantly increased risk of infection associated with TNFi monotherapy or TNFi + MTX combination therapy compared with new use of MTX without concurrent or prior biologic agent use (aHR 1.19 [95 % CI 0.72–1.94] and aHR 1.23 [95 % CI 0.69–2.17], respectively). In the hazard models, adjustment for potential confounders slightly increased the relative hazard associated with TNFi. This is owing to confounding by patient age; older age was associated both with TNFi use and with a lower baseline risk of infection. A primary hospital discharge diagnosis of infection during the baseline was strongly associated with subsequent infection after the index date compared with no infection during baseline (aHR 3.28 [95 % CI 1.44–7.48]), and this association was less strong for nonprimary hospital discharge and outpatient diagnoses (aHR 1.72 [95 % CI 1.12–2.63]). High-dose oral GC use during the baseline period was associated with infection compared with no GC use (aHR 2.03 [95 % CI 1.21–3.39]), whereas low-dose GC use was not (aHR 0.86 [95 % CI 0.50–1.46]). Sensitivity analyses that varied the outcome definition, medication risk window, duration of the baseline period to assess GC use, and duration of follow-up did not significantly change these results (data not shown). Stratified analyses of baseline GC users and nonusers did not suggest an interaction between TNFi and GCs with respect to infection (data not shown).

**Table 3:** Relative hazards of infection for tumor necrosis factor inhibitors versus methotrexate

Exposure	Comparator	Unadjusted relative hazard (95 % CI)	Adjusted^a^ relative hazard (95 % CI)
TNFi monotherapy	MTX	1.12 (0.70–1.78)	1.19 (0.72–1.94)
TNFi + MTX combination	MTX	1.21 (0.69–2.10)	1.23 (0.69–2.17)
Hospitalized infection during baseline	No infection during baseline		3.28 (1.44–7.48)
Nonprimary hospitalized or outpatient infection during baseline	No infection during baseline		1.72 (1.12–2.63)
High-dose oral GC (≥10 mg/day)	No oral GC use during baseline		2.03 (1.21–3.39)
Low-dose oral GC (<10 mg/day)	No oral GC use during baseline		0.86 (0.50–1.46)

*TNFi* Tumor necrosis factor inhibitor, *MTX* Methotrexate, *GC* Glucocorticoid

^a^Multivariable model included the following variables: new TNFi use, age, sex, asthma, baseline oral GC dose, infection during baseline period

Table 2 also shows the infection rates observed with the individual TNFi. There were no infections observed among new users of certolizumab (0.7 person-years of observation) or golimumab (3.5 person-years of observation). Compared with etanercept, adalimumab use was associated with an increased risk of infection (unadjusted HR 1.99 [95 % CI 1.03–3.87]) that persisted after adjustment for potential confounders (aHR 2.39 [95 % CI 1.21–4.72]). The characteristics of patients newly initiating etanercept and adalimumab were different: 1.0 % of etanercept new users had prior biologic agent use, compared with 28.2 % of new adalimumab users (*p* < 0.0001). Physician diagnosis codes for uveitis were observed during the 6-month baseline period in 14.0 % of new adalimumab users versus 3.1 % for etanercept (*p* < 0.0001). Further restricting the analysis to patients with no prior use of any biologic agent in the available data did not change the result (aHR 2.36 [95 % CI 1.18–4.72]). Because adalimumab received its U.S. Food and Drug Administration (FDA)-approved label for the treatment of JIA in 2008, we also restricted the comparison with etanercept to new medication initiations in 2008 and later. The resulting infection rate associated with adalimumab appeared more similar to the infection rate associated with etanercept over the same period (aHR 1.39 [95 % CI 0.62–3.19]).

Table 4 shows the relative hazards of infection for the comparison of anakinra and MTX. Anakinra was significantly associated with an increased risk of infection compared with all MTX users (aHR 3.53 [95 % CI 1.83–6.82]). High-dose oral GC use was associated with an increased risk of infection compared with no use of GCs (aHR 2.79 [95 % CI 1.35–5.78]). The increased risk of infection associated with anakinra was somewhat attenuated when the comparator cohort was restricted to MTX with SJIA (aHR 2.69 [95 % CI 0.82–8.82]).

**Table 4:** Relative hazards of infection for anakinra versus methotrexate and anakinra versus methotrexate with systemic juvenile idiopathic arthritis

Exposure	Comparator	Unadjusted relative hazard (95 % CI)	Adjusted^a^ relative hazard (95 % CI)
Anakinra	MTX	5.69 (3.30–9.81)	3.53 (1.83–6.82)
Hospitalized infection during baseline	No infection during baseline		4.81 (1.94–11.9)
Nonprimary hospitalized or outpatient infection during baseline	No infection during baseline		1.76 (0.99–3.16)
High-dose oral GC (≥10 mg/day)	No oral GC use during baseline		2.79 (1.35–5.78)
Low-dose oral GC (<10 mg/day)	No oral GC use during baseline		1.06 (0.54–2.08)
Anakinra	MTX with SJIA	3.07 (0.93–10.2)	2.69 (0.82–8.82)
Hospitalized infection during baseline	No infection during baseline		3.10 (0.74–12.9)
Nonprimary hospitalized or outpatient infection during baseline	No infection during baseline		1.76 (0.66–4.72)
High-dose oral GC (≥10 mg/day)	No oral GC use during baseline		2.05 (0.57–7.30)
Low-dose oral GC (<10 mg/day)	No oral GC use during baseline		1.59 (0.41–6.09)

*Abbreviations: MTX* Methotrexate, *SJIA* Systemic juvenile idiopathic arthritis, *mg* Milligrams of prednisone equivalents, *GC* Glucocorticoid

^a^Multivariable model included the following variables: new anakinra use, age, sex, asthma, baseline oral GC dose, infection during baseline period, cyclosporine use (time-varying).

## Discussion

In an analysis of pooled TNFi exposure, we did not observe a significant increase in hospitalized infection rates following initiation of TNFi monotherapy or TNFi + MTX combination therapy compared with initiation of MTX without prior or concurrent biologic agent use among children with JIA. These results are consistent with those of several previously published studies, while other studies have shown an increased risk of infection associated with TNFi use. One of the earliest published studies about comparative rates of serious infections in clinical practice was a prospective cohort study in which investigators found no difference in infection rates between children with JIA receiving etanercept (*n* = 103), etanercept plus MTX (*n* = 294), or MTX alone (*n* = 197) [7]. Likewise, we previously published a study using U.S. Medicaid data reporting no difference in infection rates between TNFi regardless of MTX use versus MTX use alone [4], although this study was limited by its prevalent user design necessitated by limited available data [10, 11].

More recently, reports of serious infections based on data derived from large, drug-based JIA registries have been published. Davies et al. analyzed data derived from the British Society for Paediatric and Adolescent Rheumatology Etanercept Cohort Study (BSPAR-ETN) and reported an observed increased rate of “medically significant” infections among children receiving etanercept versus MTX (aHR 2.13 [95 % CI 1.22–3.74]) [8]. However, the definition of “medically significant” was based upon the judgment of the treating physician and potentially biased the results; when the outcome was limited to infections requiring hospitalization or intravenous antibiotics, there was no increased risk of infection associated with etanercept versus MTX (aHR 1.36 [95 % CI 0.60–3.07]) [8]. Of note, the crude rate of infections requiring hospitalization or intravenous antibiotics among children receiving etanercept in that study (2.2 per 100 person-years) was similar to our observed rate of hospitalized infection (1.4 per 100 person-years), whereas the rate of medically significant infections determined by the treating physician was much higher (5.5 per 100 person-years) [8]. Additionally, the authors reported no significant increased risk of infection with etanercept plus MTX versus etanercept monotherapy, regardless of the infection outcome definition [8]. Klotsche et al. analyzed data from the German Biologics in Paediatric Rheumatology (BiKeR) registry and its follow-up registry, Juvenile arthritis Methotrexate/Biologics long-term Observation (JuMBO), and reported an increased rate of infection with etanercept versus MTX (adjusted relative risk [aRR] 2.12 [95 % CI 1.08–4.17]) but no increased rate with adalimumab versus MTX (aRR 0.88 [95 % CI 0.18–4.28]) [9]. Importantly, this study was not able to adjust for systemic GC use. Also of note, the crude infection rates for MTX (0.52 per 100 person-years) and etanercept (0.92 per 100 person-years) in this study were lower than in other published studies of serious infection in JIA [9], suggesting the possibility of underreporting of infections by physicians.

We observed that high-dose GC use during the baseline period was a significant risk factor for subsequent hospitalized infection. The results of this study showed a somewhat less extreme risk association with high-dose GCs (aHR 2.03 [95 % CI 1.21–3.39]) than did our previously published study (aHR 3.1 [95 % CI 2.0–4.7]) [4]. This different result may be due to the new-user design employed in the present analysis. In the prior prevalent-user design, the estimate for GCs may have represented both the risks attributable to GCs and those attributable to high disease activity that is frequently associated with GC use. In the present analysis, some of the confounding by high disease activity was likely diminished by the new-user design, thus attenuating the risk estimate associated with GCs [10, 11]. The authors of the BSPAR-ETN analyses reported a univariate risk associated with any baseline GC use that was similar to our study’s result (HR 2.13 [95 % CI 1.29–3.51]).

We observed a significantly increased risk of infection with adalimumab compared with etanercept. Although we restricted this analysis to patients without any use of biologic agents in the 6-month baseline period, we could not adequately adjust for the number of prior biologic agents ever used, because only 20 new users of etanercept (1 %) had any prior use of biologic agents, compared with 28 % of adalimumab users. Therefore, new users of adalimumab likely had more refractory disease and a potentially resultant increased baseline risk of infection. When we restricted the analysis to patients without any observed prior use of biologic agents, we obtained the same result, but the identification of any prior biologic agent use may have been limited by left censoring. Additionally, we observed only 12 infections among the adalimumab users, and therefore our results may be susceptible to random chance. Another consideration is that etanercept was available for several years prior to the availability of adalimumab, likely influencing prescribing patterns for both medications. When we restricted our analysis to the period after adalimumab received its FDA-approved label for JIA, the infection rates appeared more similar, but the estimate was imprecise because few infection outcomes were observed.

Interestingly, the authors of the BiKeR/JuMBO analyses reported the opposite observation. In that study, adalimumab was associated with fewer infections than etanercept (aRR 0.88 [95 % CI 0.18–4.28]), although there were only two infection outcomes in the adalimumab cohort with a resultant wide 95 % CI. Another important consideration when comparing these TNFi is the fact that adalimumab is frequently prescribed for the treatment of anterior uveitis associated with JIA and etanercept is not [14, 23]. There may be differential baseline infection risks based upon the presence of active uveitis versus synovitis. In our study, we were not able to reliably determine whether patients received TNFi for the treatment of active uveitis versus synovitis, but compared with etanercept users, the new users of adalimumab were much more likely to have diagnosis codes for uveitis during the baseline period.

In analyzing the largest published cohort, to our knowledge, of children with JIA initiating anakinra, we observed a significantly increased risk of infection compared with MTX, even with adjustment for GC dose during the baseline period. It can be assumed that most children receiving anakinra had SJIA [1, 3, 13, 14], but this could not be determined with certainty on the basis of our data, as we did not have access to medical records. When we restricted the new MTX user comparators to those very likely to have SJIA, the relative risk associated with anakinra was partially attenuated but still elevated. It must be noted that our definition of SJIA was limited by the lack of clinical data. Our definition could not be validated with the data available, and the sensitivity and specificity of the definition are unknown. For example, it is possible that a small minority of MTX users with SJIA received oral cyclosporine for treatment of skin psoriasis rather than SJIA, as suggested by the increased proportion of children with psoriasis in this subcohort compared with MTX (2.7 % versus 8.8 %). Additionally, we observed only three infections among the MTX users with SJIA; therefore, our results may be susceptible to random chance.

Previously reported rates of infection associated with anakinra are highly variable. On one hand, in one case series of 33 patients receiving anakinra [24] and one prospective cohort study of 20 newly diagnosed patients receiving anakinra without concurrent GCs [25], researchers reported no observed serious infections. On the other hand, investigators in a different published case series of 46 patients receiving anakinra as part of their initial treatment regimen reported 3 serious infections (approximate rate of 6 serious infections per 100 person-years) [26]. In addition, researchers in a clinical trial with an open-label extension period in which 22 total patients received anakinra reported 4 serious infections (approximate rate of 25 serious infections per 100 person-years) [27]. A different clinical trial of anakinra for polyarticular course JIA reported 2 serious infections among 86 total patients, but an approximate infection rate cannot be determined on the basis of the data published [28]. Similarly, published clinical trial data derived from studies of the other commercially available IL-1 inhibitors (canakinumab and rilonacept) do not allow for calculation of approximate serious infection rates for comparison [29, 30]. Looking beyond evidence in JIA, the authors of a published meta-analysis of randomized clinical trials of anakinra for adults with rheumatoid arthritis demonstrated a significantly increased risk of infection compared with placebo (odds ratio 3.40 [95 % CI 1.11–10.46]) [31].

Very little is known about background infection rates among children with SJIA compared with other categories of JIA, but it is plausible that the infection risk may be much greater. We controlled for baseline GC use to the extent possible in our study and still observed an increased risk associated with anakinra. One potential source of published evidence about the possibility of a differential baseline infection risk is a comparison of the infection rates in the two recently published clinical trials for tocilizumab, one for polyarticular JIA and the other for SJIA. In the polyarticular JIA trial, the serious infection rate was approximately 6.7 per 100 person-years during the open-label run-in portion of the trial and 3.1 per 100 person-years during the remainder of the study [32]. In the SJIA trial, the rate of serious infection was approximately 17 per 100 person-years [33], suggesting an increased risk of infection in children with SJIA that is independent of biologic agent use. Of course, these studies are not directly comparable, because the patients in the SJIA study received higher doses of tocilizumab and GCs. We attempted to control for the effects of SJIA by restricting the MTX comparator group to children with likely SJIA, but residual confounding by disease severity and disease activity was likely present, as we could not reliably assess these factors using administrative claims data.

It is notable that our study was limited by the data source. We used a new-user design to control for arthritis disease activity at the time of newly starting a medication [11] because disease activity is known to be associated with an increased risk of infection in adults with rheumatoid arthritis [34]. Nevertheless, administrative claims do not contain clinical data, and residual confounding by disease activity was possible in our results and could not be further evaluated in this study.

## Conclusions

We did not observe an increased risk of infection with TNFi monotherapy or TNFi + MTX combination therapy compared with MTX alone. Recent infection and high-dose oral GC use were important risk factors for hospitalized infection. Adalimumab was associated with an increased risk of infection compared with etanercept, but this result may be confounded by disease severity. Anakinra was associated with increased risk of infection compared with MTX. More studies are needed to elucidate the risks of infection associated with SJIA and its treatment.

## Abbreviations

aHR: Adjusted hazard ratio
aRR: Adjusted relative risk
BiKeR: Biologics in Paediatric Rheumatology
BSPAR-ETN: British Society for Paediatric and Adolescent Rheumatology Etanercept Cohort Study
FDA: U.S. Food and Drug Administration
GC: Glucocorticoid
ICD-9: International Classification of Diseases, Ninth Revision
IL: Interleukin
JIA: Juvenile idiopathic arthritis
JuMBO: Juvenile arthritis Methotrexate/Biologics long-term Observation
MAX: Medicaid Analytic eXtract
MTX: Methotrexate
N/A: Not applicable
SJIA: Systemic juvenile idiopathic arthritis
TNFi: Tumor necrosis factor inhibitor
